# Is the Philippines ready for the large-scale cultivation of *Bt* cotton?

**DOI:** 10.1080/21645698.2025.2499997

**Published:** 2025-05-02

**Authors:** Rosalyn B. Angeles-Shim, Georgina V. Vergara

**Affiliations:** a Department of Plant and Soil Science, Davis College of Agricultural Sciences and Natural Resources, Texas Tech University, Lubbock, TX, USA; b Institute of Crops Science, College of Agriculture and Food Science, University of the Philippines, Los Baños, Philippines

**Keywords:** bollworm, capacity building, *Gossypium hirsutum*, pest resistance, transgenic plants

## Abstract

The Philippine Bureau of Plant Industry released on August 24, 2023, a biosafety permit approving the commercial propagation of *Bt* cotton in the country. *Bt* cotton is genetically engineered to have built-in resistance to bollworm, the most destructive pest of the cotton plant. With the issuance of the permit and after more than two decades of economic slump, the Philippines is now gearing towards a renaissance of the cotton industry. But is the Philippines ready to implement the agri-technological innovation that is the *Bt* cotton? This paper provides insights into the promise of the proposed large-scale adoption of *Bt* cotton, as well as a commentary on the potential challenges that can impede the long-awaited revival of the cotton industry in this island country. Both challenges and potential solutions are presented in the context of institutional, financial and technical capacity of the Philippines to support the wide scale *Bt* cotton initiative.

## Introduction

On August 24, 2023, fourteen years after the Philippine Fiber Industry Development Authority (PhilFIDA) launched the *Bt* cotton project in 2009 in a bid to revitalize the country’s dying cotton industry, the Philippine Bureau of Plant Industry finally issued a biosafety permit allowing the commercial propagation of a hybrid *Bt* cotton (*GFM cry1A)* in the country.^1^ The Philippines now joins the ranks of a few nations in Asia Pacific, including India, China, and Pakistan, that are commercially cultivating genetically modified cotton.^2^

Cotton is an indigenous fiber crop that has been traditionally cultivated as an integral part of the Philippine weaving industry. The native cultivars grown in the Philippines are of the allotetraploid island species *Gossypium barbadense* (i.e. Pima cotton), which produces exceptionally high-quality fibers. Despite its national and economic significance, commercial cultivation of cotton began only in the early 1970s and flourished until the early 1990s. At the peak of cotton production in the country, the local industry was able to provide at least 25% of the nation’s demand for this natural fiber. In the early 2000, however, cotton production started to plummet, with the area planted to the crop dwindling from approximately 38,000 to a measly 4,166 hectares nationwide. This significant downturn was driven primarily by the high costs of production (US$ 405/ha average) and relatively low returns (US$ 104.39/ha average). The poor profitability of the cotton enterprise, which averages only 26% in return of investments, led to farmers losing interest in growing the crop.^3,4^

Analysis of the productivity of cotton farming following rice and corn planting seasons in the islands of Luzon and Mindanao in the Philippines, respectively, identified labor and agronomic inputs (i.e. irrigation, fertilizers and pesticides) as the major components of capital outlay for cotton production. Among these, the costs of pesticide application to control major cotton pests such as cotton bollworm (*Heliothis armigera*) and pink boll weevil (*Anthonomus grandis*) accounted for 30–43% of the total production costs.^4,5^

Cotton bollworm is a destructive pest of cotton worldwide and was the key driver to the decline of the cotton industry in the Philippines. Bollworms attack cotton plants as early as the vegetative stage, feeding on leaf terminals, fruit buds, flowers, and developing bolls. Philippine cotton cultivars lack natural mechanisms for defense against bollworms and hence is very susceptible to damages from the pest.^6,7^ Local farmers would normally apply full doses of synthetic chemicals 8–10 times within a growing season to protect traditional cotton cultivars from bollworms. Despite significant investments in chemical control strategies, farmers still lost 30–65% of their potential yield due to insect damage. The farmers’ losing battle against bollworms eventually led to the abandonment of cotton cultivation in exchange for more profitable cash crops like rice, corn, and even seasonal vegetables. As the land acreage dedicated to cotton production steadily dropped, the Philippine cotton industry dived into an economic slump that has lasted to date. With local production costing more than the imported cotton, the textile industry turned to importing the fiber commodity. In the last 20 years, the Philippines has imported a total of 472,000 bales of cotton. In 2022 alone, the Philippines imported US$ 145.54 million worth of cotton from its neighbors, Vietnam, China, and India to meet the demands of the local textile industry.^6^

## The Promise of *Bt* Cotton to Reinvigorate the Philippine Cotton Industry

To reinvigorate cotton farming and local weaving in the Philippines and at the same time wean the country from its decades-long dependency on imported cotton, PhilFIDA initiated the *Bt* cotton project in 2009. The program advocates for the use of biotechnology, specifically the adoption of genetically modified *Bt* cotton as an alternative to the local cultivars traditionally grown by Filipino farmers. *Bt* cotton contains the *Bt* fusion *cry1A* gene that was synthesized from the cry1Ab and cry1Ac protein templates of the soil bacterium, *Bacillus thuringiensis*. It produces toxic proteins that damage the gut lining of newly hatched bollworms. The toxin specifically targets cotton bollworm and has no known adverse effects on non-target organisms including beneficial insects.^6,7^ With bollworm resistance already genetically engineered in *Bt* cotton, its cultivation promises to cut down the costs of pesticides that have been traditionally used to manage damages by the pest on susceptible, native cultivars. More importantly, it has the potential to increase current yields from 1–2 tons to 3 tons per hectare. These translates to higher profits to farmers and to a more sustainable enterprise for the local textile and indigenous weaving industry. Farmers who pioneered in planting the hybrid *Bt* cotton in the northern province of Ilocos Norte reported a gross income of Php 350,000 (approximately US $6,245) from 2.5 ha of land. This is more than double the profit that can be gained from planting rice.^8,9^

The implementation of the *Bt* cotton project in the Philippines was initiated by purchasing six commercial varieties of hybrid *Bt* cotton produced by Nath Seeds/Global Transgenes Ltd in India using Chinese technology.^7^ All the transgenic hybrids were of the *G. hirsutum* genetic background. The acquisition of the *Bt* biotechnology was followed by a breadth of biological safety evaluation that included greenhouse experiments in 2009–2010, confined field testing in 2010–2011 and multi-location trials in the provinces of Ilocos Norte, Pangasinan, Sarangani, Cotabato and Polomolok in 2012–2015. In every level of evaluation, the hybrid *Bt* cotton perceptibly and consistently performed better than the local varieties in terms of pest resistance and yield. In a field trial in one of the PhilFIDA stations in Ilocos Norte, the hybrid *Bt* cotton varieties NECH 21 and NECH 34 demonstrated superiority in their resistance to bollworm compared to the non-*Bt* native variety UPL-C_2_ [personal communication; Cacayorin, ND; PhilFIDA Unit Head].

Following the approval of the commercial propagation of *Bt* cotton, the Philippines is gearing to promote the expansion of cotton cultivation in the country. Technological demonstrations (techno-demos) that aim to highlight the benefits of planting *Bt* cotton have been initiated in Ilocos Norte and are currently underway in the provinces of Pangasinan, Nueva Ecija, Sarangani and South Cotabato. The techno-demos that will occupy 40 one-hectare farms across the country will serve as a forum to educate farmers, other stakeholders within the cotton value chain (i.e. ginners, classers, prepared cotton traders/merchants, textile manufacturers or weavers, garment manufacturers, retailers, consumers) and government officials on the science and merits of *Bt* technology.^2^

Aside from extension efforts, research that will integrate *Bt* technology into local cotton cultivars will also be initiated during the first year of *Bt* cotton commercialization.^2^ The *Bt* transgene in the genetic background of native cultivars is expected to produce a cotton crop with stable resistance against bollworm and high-quality lint that is preferred by the local weavers. This will not only strengthen the Philippine cotton industry and make it competitive against foreign markets but will also allow the preservation of heirloom cotton cultivars that have been grown by Filipino farmers for centuries.

But does the Philippines have the institutional, financial and technical capacity to support the large-scale cultivation of *Bt* cotton?

## Challenges in the Widespread Adoption of *Bt* Cotton Technology in the Philippines

The path to the successful acquisition of legal permission to commercially grow *Bt* cotton in the Philippines has been long and arduous. It is therefore critical that cotton production, research and extension systems that can support *Bt* cotton cultivation be established for the widespread acceptance and adoption of this agricultural innovation. Ultimately, the extent by which production, research and extension efforts can build the technical and institutional capacities of stakeholders within the cotton value chain will determine the successful revival of the cotton industry in the Philippines.

From a production standpoint, the most obvious requirement to ensure the overall success of this agricultural innovation is through the implementation of the best agricultural practices that will ensure optimum yield in farmers’ fields. This can be reasonably met by disseminating information to farmers regarding the agronomic practices implemented by PhilFIDA during the initial performance evaluations of the *Bt* cotton varieties in greenhouses, confined fields and multiple field locations in the Philippines. Any necessary improvements in cultural practices could be based on farmers’ experiences growing *Bt* cotton in their own fields. This will require close monitoring of farmers’ practices and collection of relevant agronomic data on production inputs and outputs to aid in the improvement of existing agricultural practices for *Bt* cotton production.

Equally important are potential challenges related to the existing technological framework and infrastructure, technology transfer as well as social acceptance of the technological innovation that can hinder the widespread adoption of *Bt* cotton in the Philippines. Some of these challenges, which are presented in the succeeding paragraphs, can be gleaned from experiences of the Philippines with similar biotechnologies and of other nations that adopted *Bt* cotton. These potential obstacles need to be identified as they relate to the Philippine cotton industry and used as a basis to create action plans that can be implemented to curtail or address such obstacles.

*Technological framework and infrastructure*. The Philippines has traditionally grown cotton varieties of the *G. barbadense* background although in recent years, cultivars of *G. hirsutum* with resistance to specific biotic stresses have been developed and released for cultivation (Table 1). In contrast, the *Bt* cotton from India is an intraspecific hybrid in the genetic background of *G. hirsutum*. Both *G. barbadense* and *G. hirsutum* thrive under long-season cultivation under warm climates (i.e. 21–30°C), with the former requiring longer growing season under little or no rainfall for optimum fiber maturation. Both species are allotetraploids although they evolved independently and were domesticated in diverse geographic regions. *G. hirsutum* has high yield potential but moderate fiber quality whereas, *G. barbadense* has moderate yield potential but produces exceptional fiber quality (i.e. longer and stronger staples) that caters to specialty textile producers.^10^ Given the significant differences in fiber quality produced by the local and introduced *Bt* cotton, the technological framework and infrastructure for downstream processing of lint including ginning, spinning, milling and application of prepared cotton need to be modified, upgraded, and updated to meet the requirements for processing the shorter *Bt G. hirsutum* fibers. These modifications will require accompanying training on the appropriate processing, grading and other downstream applications of the shorter *G. hirsutum* fibers.

**Table 1.** Current recommended varieties of cotton in the Philippines (2017).

Variety	Pot Yield	Lint Recovery (%)	Fiber properties			Biotic Stress Resistance
			Length (mm)	Strength (g/tex)	Fineness (µg/in)	
Upl-C2*	2.10	38.90	29.80	19.44	5.10	leafhopper (MR), bacterial blight (R)
CRDI-1*	2.12	40.80	28.90	19.43	4.31	leafhopper (HR), damping off (MR)
CRDI-2*	2.13	41.00	27.61	19.61	4.35	damping off (R), boll rot (R)
PSB-Ct_8_*	2.23	39.76	30.39	19.45	4.58	damping off (R), Fusarium wilt (R), leafhopper (MR)
NSIC-Ct11*	2.44	40.76	28.72	18.63	4.74	leafhopper (R), damping off (R)
NSIC-Ct12**	2.52–3.36	36.09	30.07	20.15	3.82	leafhopper (R)
NSIC-Ct13**	2.43	37.17	27.91	20.41	4.12	leafhopper (HR), damping off (R)
PSB-Ct9*	1.43	26.91	39.19	28.49	2.5	leafhopper (MR), damping off (HR/R)
PSB-Ct10*	1.30	28.68	37.04	27.99	<2.5	leafhopper (HR), damping off (HR/R)

*purelines.

**hybrid.

MR = moderately resistant; R = resistant; HR = highly resistant.

In line with expected large-scale operations, the cotton sector should also prepare for the possibility of mechanization to facilitate expansion of *Bt* cotton cultivation. Manual picking will provide job opportunities for more people but will not be practical in view of the targeted widespread *Bt* cotton farming that meets the demands of the local weaving industry.

Incidentally, the short- and long-term impacts of the large-scale adoption of *Bt* cotton on the indigenous weaving industry should also be considered. Promotion of the widespread cultivation of *Bt* cotton aims not only to reinvigorate cotton farming in the Philippines but also to support and sustain the growth of the local weaving industry by ensuring the availability of locally produced raw fiber materials. For generations, Philippine indigenous weavers have spun the exceptionally long fibers of *G. barbadense* into products that include blankets and native clothing using traditional handlooms. While these handlooms may have been efficient in spinning long staples, they might not be as effective in weaving the shorter fibers of *Bt G. hirsutum*. With the expected abundance of shorter fibers from *Bt G. hirsutum* farming, indigenous weavers would require both technical and financial assistance to update their handlooms and make it more suitable for processing shorter staples or shift from using handlooms to simple but more contemporary machines appropriate for processing shorter staples. A more important issue that needs to be addressed is the strong preference of local weavers for high-quality fibers from the local *G. barbadense* varieties which can bias them against weaving *G. hirsutum* staples. To ensure that *Bt G. hirsutum* will be patronized and used for weaving, local weavers should be presented with product alternatives that require moderate quality fibers and be assured of a market for such products. Otherwise, the non-acceptance of *G. hirsutum* staples by the indigenous weavers could greatly undermine the very purpose of *Bt* cotton adoption in the Philippines and lead to an outcome similar to what happened in Burkina Faso. In 2000, Monsanto introduced the *Bt* cotton Bollgard II in Burkina Faso to address the bollworm problem that was threatening cotton production in the country. The cultivation of Bollgard II significantly improved yields and increased farmers’ profits but the quality of fibers produced by the transgenic plant failed to deliver the quality of the homegrown crop. The locals did not want to trade off quality over quantity and eventually abandoned the cultivation of Bollgard II.^11^

*Technology transfer*. The hybrid *Bt* cotton seeds that were grown for the series of biological safety evaluations in the Philippines has been imported from Nath Seeds/Global Transgenes Ltd in India.^7^ This presents a problem for low-income farmers because of the general lack of credit facilities that can provide them with the initial, let alone yearly capital for *Bt* cotton farming. In this regard, the goal of PhilFIDA to integrate *Bt* technology in local cotton varieties in the Philippines is a right step toward releasing Filipino cotton farmers from their dependence on big Biotech companies for seeds. The transfer of the *Bt* transgene to local cotton cultivars through traditional breeding, however, will not be straightforward and will require experiential knowledge in cotton genetics and breeding. *G. barbadense* and *G. hirsutum* are amenable to artificial hybridization that produce fertile F_1_s. The subsequent selfing of the F_2_s, however, produces plants with genomic constitutions that are biased toward one parent. This preferential chromosome transfer is attributed to large-scale inversions in the chromosomes of *G. barbadense* and *G. hirsutum* genomes that limit genomic exchanges in the inverted regions, as well as in the loci adjacent to the inversions.^12–14^ Standards for genotyping and phenotyping breeding lines possessing the transgene will have to be optimized to ensure the successful introgression of *Bt* in the background of local cultivars. When Bollgard was introduced to Burkina Faso, Monsanto consented to breed the *Bt* transgene into the background of the local cultivar. Testing in 2006–2008 of early backcrosses carrying the transgene, however, showed reduction in the Burkinabe *Bt* fibers by 0.88–2.41 mm. By 2014–2015, the Burkinabe *Bt* cotton is producing fibers that are 2.29 mm shorter than the conventional cultivars. The lack of technical expertise and capacity to transfer the trait in the genetic background of native varieties resulted in the failure of the technology transfer that could have improved the cotton industry in the country.^11^

*Social acceptance of the technology*. Perhaps a bigger challenge to the success of large-scale cultivation of *Bt* cotton is the attitude toward this technological innovation of critical stakeholders in the cotton industry which include not only the weavers but also the farmers. Farmers are key players upstream of the cotton value chain and are usually the most economically underserved. In the Philippines, most farmers grow crops in smallholdings that provide only the needs of the farmers themselves and their families. For *Bt* cotton to be widely accepted for cultivation, it should primarily ensure farmers’ income.

There has been no shortage of expertise in the Philippines to develop genetically modified (GM) crops, nor actual GM food products from both private and public crop improvement programs. The *Bt* eggplant is the first biotech product from the Philippines. It was developed by plant breeders from the Institute of Plant Breeding of the University of the Philippines at Los Baños, Laguna using a *35S CaMV-cry1Ac* transgenic event in eggplant that was developed by the Maharashtra Hybrid Seeds Co. Pvt. Ltd. (Mahyco). The *Bt* eggplant produces a protein from the soil bacterium *B. thuringiensis* that makes it resistant to shoot borer, the most destructive pest of eggplant.^15^ Golden rice on the other hand, was developed by the International Rice Research Institute with headquarters in the Philippines to address the issue of vitamin A deficiency in children and pregnant women worldwide.^16^ The Golden rice has been genetically modified to produce β-carotene, a plant pigment that the human body converts into vitamin A.^17^ To date, however, only the genetically engineered *Bt* corn has been successfully grown in the country.^18^ Golden Rice and *Bt* eggplant were initially approved for commercial propagation in 2021 and 2022, respectively. These decisions were overturned by the Supreme Court on April 17, 2023, and was upheld by the Court of Appeals exactly a year after.^19,20^ The basis of the decision came from arguments from environmentalists regarding the potential of GM plants to cause genetic erosion and contaminate other crops in conjunction with the scientific community’s failure to provide substantial evidence to counter such claims. The decision was celebrated not only by environmentalists but surprisingly, by Filipino farmers. The farmer’s stand against GM crops does not necessarily align with those of the environmentalists but is rooted instead on a deep-seated distrust of previous governments and major seed companies that historically tried to gain a top-down control of agriculture. On the heels of the court decision to ban *Bt* eggplant and Golden rice, it is vitally important to educate stakeholders in the cotton value chain regarding the null impact on the environment and human health of *Bt* cotton. More importantly, the farmers should also be informed about the potential economic benefits of cultivating *Bt* cotton.

## Conclusion

The Philippines has invested a tremendous amount of time and resources in support of a cotton industry revival that depends on the large-scale cultivation of varieties with genetically engineered resistance against bollworm. The adoption of *Bt* cotton technology in the country following the official approval of its commercial cultivation promises a sustained growth and development of the cotton sector that has long suffered an economic slump. For the economically underserved stakeholders of the cotton value chain, this initiative is a promise of increased income toward improvements in quality of life. As a technological novelty however, the adoption and large-scale cultivation of *Bt* cotton are likely to face challenges related to the general lack of technical and institutional capacity to support production, research, and extension efforts for *Bt* cotton. To unlock the full potential of *Bt* cotton technology in the Philippines toward the sustained growth and productivity of this agricultural sector, the technical and institutional capacity of key stakeholders within the cotton value chain to promote, sustain, and strengthen the widespread acceptance, growth, and development of *Bt* cotton production needs to be established.
